# Nanoelectromechanical modulation of a strongly-coupled plasmonic dimer

**DOI:** 10.1038/s41467-020-20273-2

**Published:** 2021-01-04

**Authors:** Jung-Hwan Song, Søren Raza, Jorik van de Groep, Ju-Hyung Kang, Qitong Li, Pieter G. Kik, Mark L. Brongersma

**Affiliations:** 1 Geballe Laboratory for Advanced Materials, Stanford University, Stanford, CA 94305 USA; 2 Department of Physics, Technical University of Denmark, DK-2800 Kongens Lyngby, Denmark; 3 CREOL, The College of Optics and Photonics, University of Central Florida, Orlando, FL 32816 USA; 4 Present Address: Van der Waals–Zeeman Institute for Experimental Physics, Institute of Physics, University of Amsterdam, Amsterdam, Netherlands

**Keywords:** Nanophotonics and plasmonics, Micro-optics

## Abstract

The ability of two nearly-touching plasmonic nanoparticles to squeeze light into a nanometer gap has provided a myriad of fundamental insights into light–matter interaction. In this work, we construct a nanoelectromechanical system (NEMS) that capitalizes on the unique, singular behavior that arises at sub-nanometer particle-spacings to create an electro-optical modulator. Using in situ electron energy loss spectroscopy in a transmission electron microscope, we map the spectral and spatial changes in the plasmonic modes as they hybridize and evolve from a weak to a strong coupling regime. In the strongly-coupled regime, we observe a very large mechanical tunability (~250 meV/nm) of the bonding-dipole plasmon resonance of the dimer at ~1 nm gap spacing, right before detrimental quantum effects set in. We leverage our findings to realize a prototype NEMS light-intensity modulator operating at ~10 MHz and with a power consumption of only 4 fJ/bit.

## Abstract (web summary)

Squeezing light into a nanometer gap offers strong light–matter interaction. Here, the authors develop a nanoelectromechanical system to dynamically control the gap of a plasmonic dimer at nanometer scale, enabling the realization of a light-intensity modulator that operates at high speed and with a low power consumption.

## Introduction

The plasmonic dimer, comprised of two closely-spaced metal nanoparticles, has served as the prototypical system to study near- and far-field optical coupling of plasmonic structures^1–4^. The understanding and engineering of such coupling is central to the design of metamaterials and nanophotonic devices. Most notably, the overlap of the near-fields in closely-spaced metal particles can be used to controllably hybridize individual plasmon modes. This can lead to a desirable redistribution of optical fields and an intense light concentration^5–7^. The extreme light confinement renders the dimer’s optical response very sensitive to minute changes in gap size^8^ and facilitates the observation of a variety of intriguing quantum effects^9–13^. These findings naturally prompt the fundamental questions to what extent fields can be concentrated and resonances be tuned^14^. Classical treatments predict singularities in the achievable field enhancement and frequency shifts^8,15^, suggesting an unparalleled opportunity to enhance light-matter interaction and create tunable devices. However, theory and experiments have also demonstrated that quantum mechanical tunneling of charge across the gap eliminates such desirable singular behavior when gap sizes are reduced to about 0.5 nm^3,15,16^. Such gaps are still at least 2 orders of magnitude smaller than the wavelength of light and these works indicate that the fundamental size limit of electro-optical modulators may need to be re-evaluated. Currently, the highest performance modulators are many microns in size as they leverage weak electro-absorption or electro-refraction effects and rely on interferometric approaches to achieve switching. Because of their large size, they are also very power hungry^17^. However, new materials exhibiting unity-order index changes^11,12,18–20^ and new device physics concepts employing plasmonics^21–23^, electrochemistry^24–26^, and/or nanomechanics^27–29^ are now opening new pathways for scaling. The availability of low-power, electrically-tunable optical elements with a deep-subwavelength footprint would have a transformative impact on the development of dynamic metasurfaces^30,31^, optical communication^17^, optical neural networks^32^, and quantum information processing^33^.

Here, we design and implement a nano-electromechanical system (NEMS) to dynamically modulate the gap of a dimer at the ultimate, atomic scale (~1 nm), which allows operation at fundamentally-limited optical sensitivities and achievement of low-power (~1 fJ/bit), high-speed (~10 MHz) manipulation of optical signals. To aid the design and verify the operation of this modulator, we capitalize on recent advances in electron energy loss spectroscopy (EELS). This technique measures the energy loss probability of swift electrons as they interact with materials and structures. It can be employed to map optical modes of nanoscale plasmonic resonators with unparalleled spatial resolution and has provided valuable spectral information on the optical resonances of static plasmonic elements^18–23^. In this work, we dynamically follow the modal hybridization of a single, dimer element upon electromechanical actuation inside a transmission electron microscope (TEM).

## Results

### Nanoscale electromechanical control over a plasmonic dimer

**Fig. 1:**
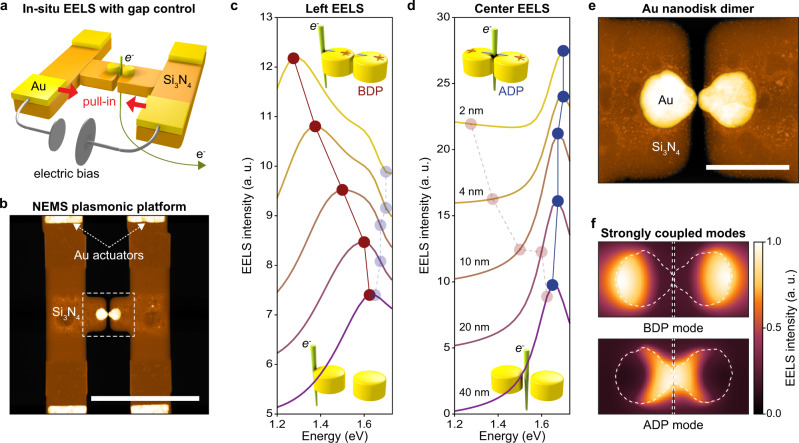
Nanoelectromechanical system (NEMS) with a plasmonic dimer. **a** Schematic of the device showing how an external DC bias applied to Au actuators can electrostatically pull two Si_3_N_4_ beams together and reduce the spacing between two Au nanoparticles. **b** False-colored STEM image of the fabricated dimer (white/yellow) and cantilevers (brown). White, dashed rectangle indicates the location of the dimer. Scale bar: 1 μm. **c, d** Simulated EELS spectra as a function of gap size for the electron beam incident on the left edge (**c**) and the center (**d**) of gold nanodisk dimer. We apply an increasing 1 step (5 step) offset values to each of the left (center) EELS intensity spectra for improved visibility. The red and blue symbols indicate the location of BDP and ADP modes, respectively. **e** Magnified false-colored STEM image of gold nanodisk dimer. The diameter and thickness of gold nanodisk are designed to be 100 nm and 50 nm, respectively. Scale bar: 200 nm. **f** Simulated EELS spatial profile of BDP and ADP modes for the gap size of 2 nm. White dashed contour extracted from STEM image (Fig. 1e) has been taken into account in the EELS simulation.

Figure 1a, b show schematic and TEM images of a NEMS that can dynamically tune the gap of a gold (Au) nanoparticle-dimer. The device was fashioned inside a thin Si_3_N_4_ TEM window (Methods). The side-by-side arrangement of the particles allows convenient monitoring of the spatial and spectral properties of the plasmonic modes at different gap sizes. Simulations of EELS spectra (Fig. 1c, d) for this structure illustrate how the interparticle coupling evolves with decreasing gap size. The EELS simulations are performed with the Metallic NanoParticles Boundary Element Method (MNPBEM) toolbox for MATLAB, which solves Maxwell’s equations in the presence of an electron beam (see Methods for details). The fundamental dipolar modes of the individual nanoparticles couple to form two new eigenmodes with different modal symmetry: the bonding dipole (BDP) and the anti-bonding dipole (ADP) mode (see Supplementary Note 1). For gap sizes below 10 nm, the interparticle coupling is sufficiently strong to spectrally separate the two modes. The BDP mode continues to red-shift, ultimately at astounding rates over 100 meV for each nanometer of movement. Based on their different modal symmetries, the BDP and ADP are most effectively excited and probed in different spatial locations. This can be seen in the simulated EELS maps for these modes of a fabricated dimer (Fig. 1e) in the strong coupling regime (see Fig. 1f). This facilitates modal identification in experiments.

**Fig. 2:**
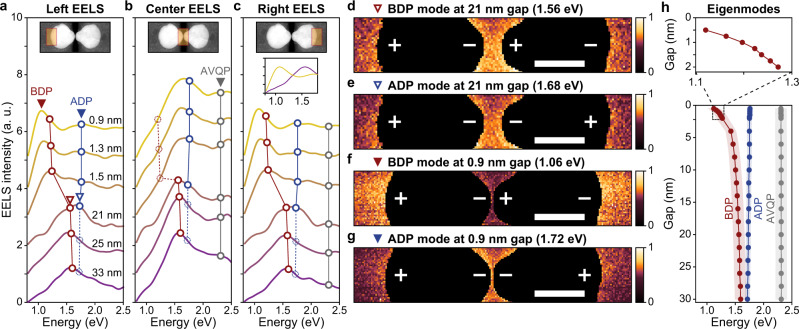
Evolution of plasmon resonances from weak to strong coupling. **a**–**c** EELS spectra as a function of the gap size while scanning the electron beam over a rectangular region on the left (**a**), center (**b**), and right (**c**) part of the gold nanodisk dimer, as indicated by the orange-shaded regions in the inset STEM images. The inset spectra in Fig. 2c directly compare the EELS intensity for 33 nm and 0.9 nm gap sizes. Overlaid red, blue, and gray circles and trend lines indicate the peaks from numerically simulated eigenmode energies for the BDP, ADP, and AVQP modes, respectively (Fig. 2h). We offset each consecutive EELS intensity spectrum by equidistant, 1-step-values for improved visibility. **d, e** Spatial EELS maps of the BDP and ADP modes for a 21-nm-gap size. Scale bars: 50 nm. **f, g** Same EELS maps for a 0.9 nm gap size. Scale bars: 50 nm. **h** Evolution of the resonant photon energies of BDP, ADP, and AVQP modes as predicted by eigenmode solutions. The shaded regions indicate the linewidth for each mode. The inset highlights the region in which the BDP strongly red-shifts and the numerically calculated resonance sensitivity to the gap size for ~1 nm.

Figure 2a–c shows experimentally obtained EELS spectra at different gap sizes and taken with the electron beam in three different locations. In each location, the EELS signal provides a direct, quantitative measure of the excitation efficiency of a plasmon mode at a certain energy. With increased bias, we continuously decrease the gap size from 33 nm in the as-fabricated device down to 0.9 nm. For the 33 nm gap, all three EELS spectra show a peak at 1.56 eV, in agreement with the simulated energy of the BDP. Increasing the bias to 30 V reduces the gap to 21 nm. This red-shifts the BDP to 1.51 eV and causes the emergence of a new peak at 1.68 eV where the ADP mode is expected. Due to a mechanical instability in the cantilever system, a jump in the gap size from 21 nm to 1.5 nm occurs for biases exceeding 40 V (see Supplementary Note 2). As a result, the BDP abruptly shifts by 0.30 eV from 1.51 eV to 1.21 eV. This sudden transition occurs when the attractive electrical forces exceed the restoring spring forces of the cantilever system, much akin to the snap-to-contact seen in atomic-force microscopy. Importantly, we find that in this near-contact state the Au particles are still physically separated by a thin, lossy-dielectric spacer layer, as demonstrated below. By increasing the DC bias, we can controllably compress the spacer to further reduce the gap size down to 0.9 nm. In this regime, the BDP resonance energy acquires a large shift of 0.15 eV from 1.21 eV to 1.06 eV as the gap size changes by only 6 Å, i.e. a remarkable sensitivity of 250 meV/nm. A direct comparison of the EELS spectra taken for gap sizes of 33 nm and 0.9 nm (inset to Fig. 2c) emphasizes that 30% of the entire spectral shift seen in reducing the gap is acquired in the last 6 Å of movement. The EELS spectra taken with the electron beam in the gap region (Fig. 2b) shows the ADP resonance peak most clearly. Decreasing the gap size from 1.5 nm to 0.9 nm gap size also causes a small blue-shift of the ADP from 1.69 eV to 1.79 eV. The third resonance near 2.40 eV is identified as the anti-bonding vertical quadrupole (AVQP) mode (see Supplementary Note 3), which does not shift noticeably with gap size.

In comparing the EELS spectra obtained from the outer edges (Fig. 2a, c) with those from the center (Fig. 2b), we note two important differences. First, for gap sizes smaller than 1.5 nm the BDP peak is strongly suppressed in EELS spectra taken from the central region of the dimer as compared to the spectra taken at the left/right outer edges of the dimer. Second, for gap sizes smaller than 1.5 nm the ADP peak becomes more pronounced in the spectra taken in the central region. Both observations can be understood by considering the charge symmetries in the mode profiles. To clarify this, we leverage the high spatial resolution of EELS to map the spatial modal profiles at different resonance frequencies. We compare the EELS maps—corresponding to the excitation efficiency maps—for a 21-nm-gap size (Fig. 2d, e) with those for a 0.9-nm-gap size (Fig. 2f, g). At a 21-nm-gap size, the interparticle coupling is weak and the BDP and ADP modes spectrally overlap. As a result, these modes cannot be mapped independently and the spatial profiles taken at the relevant resonance frequencies appear similar. For the 0.9-nm-gap size, on the other hand, the interparticle coupling becomes very strong and this produces well-separated resonance frequencies. Here, the local electron beam excitation efficiency is strongly governed by the parity of the modes. Electrons incident near the center of the gap tend to induce surface charges with identical sign, which makes it improbable to excite the anti-symmetric charge distribution across the gap that are characteristic for the BDP and therefore results in relatively low signal from the gap region in Fig. 2f. In contrast, the electron beam effectively excites the symmetric gap charge distributions of the ADP resulting in the comparatively large signal from the gap region in Fig. 2g.

We employ a numerical analysis of the dispersive behavior of various modes in gold dimer structures^34^ (see Supplementary Note 1) to verify our modal identification (Fig. 2h). The simulated spectral position and dispersion with gap size are in good agreement with measured spectra (Fig. 2a–c) for gap sizes from 30 nm to 1.5 nm. The simulated resonance frequencies of the BDP mode start to deviate from the measured peak locations when the gap size becomes smaller than 1 nm, showing a weaker sensitivity (170 meV/nm). For such small gaps, the resonance frequency is extremely sensitive to the exact shape of the particles. Simulations reveal that fabrication-induced irregularities of the nanodisk shapes and a non-uniform gold thickness affects the plasmon coupling and can explain the enhanced sensitivity seen in our experiments (see Supplementary Note 4). At smaller gap distances quantum effects are known to turn red-shifts into blue-shifts^3,15,16^ and this would reduce the modulation depth of a modulator. As the focus of our current work is on modulation of optical signals, we have not tried to further reduce the gap size and instead directed our attention to achieving high-speed modulation of optical signals.

**Fig. 3:**
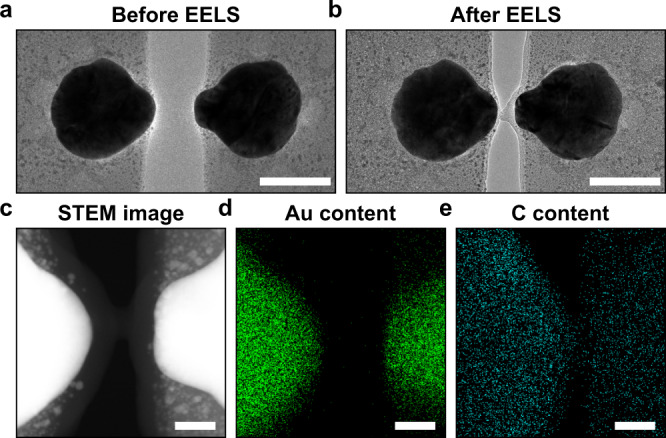
Formation of a conductive, flexible gap bridge through EBID. **a, b** TEM image taken before EELS (**a**), TEM image taken after EELS (**b**). Scale bars: 100 nm. **c**–**e** STEM image of EDS inspect region (**c**), EDS map for gold content (**d**), EDS map for carbon content (**e**). Scale bars: 10 nm.

In the device discussed in Fig. 2, we could not further reduce the gap size below 0.9 nm, even for an external DC bias of 100 V. Comparing TEM images of the sample before (Fig. 3a) and after the EELS measurements (Fig. 3b) clearly shows the presence of new, material deposits inside the gap. Energy-dispersive X-ray spectroscopy (EDS) confirms the presence of carbonaceous material in the gap (Fig. 3c–e). It is well-established that such deposits can form due to the electron beam induced deposition (EBID) of adsorbed hydrocarbon molecules^35,36^. The optical properties of the EBID material are those of a lossy dielectric and have previously been obtained by spectroscopic ellipsometry^37^. The properties are used in our optical simulations^37^. The formation of the thin dielectric spacer allows repeatable mechanical tuning even at ~1 nm gap size by preventing the two nanodisks from touching and coalescing, which is essential for light intensity modulation.

### Realization of a nanoscale electromechanical modulator

**Fig. 4:**
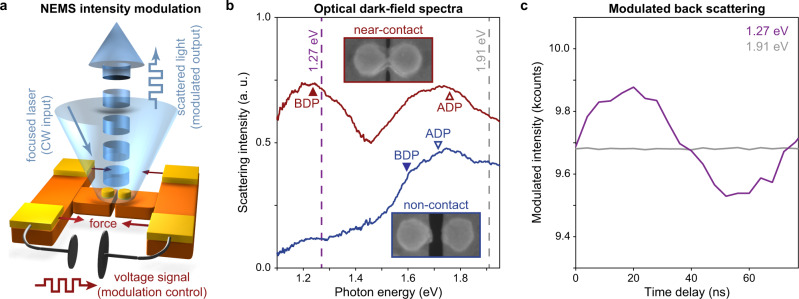
Dynamic light intensity modulation in a nanoelectromechanical device. **a** Schematic of the light intensity modulation experiment. **b** Resonant dark-field scattering spectra taken from the dimer when there is only a 1.5-nm-thick EBID spacer layer separating the two Au nanoparticles (red curve) and for the case of a 30 nm spacing (blue curve). For clear comparison, two different offsets of 0.15 and −0.35 are applied to the red and blue spectra, respectively. The magenta and gray dashed lines indicate the photon energies of the CW excitation on resonance (1.27 eV) and off resonance (1.91 eV), respectively. The triangular symbols indicate the resonance frequencies of each mode at 1.5 nm (red) and 30 nm (blue) gap sizes. **c** Time-modulated intensity signal from dimer with the 1.5-nm-EBID spacer as measured by time-correlated single-photon counting. Line plots indicate the averaged intensity in each 4-ns-wide time bin (1000 data points within each 4 ns bin). To allow easy comparison, an offset of 8 kcounts is applied to the modulated intensity for 1.91 eV laser illumination.

Capitalizing on the high sensitivity of the BDP resonance to gap size, we aim to demonstrate an ultra-compact NEMS light-intensity modulator. To this end, we created a new dimer device that can offer a ~4 fJ/bit modulation energy according to our electromechanical design (see Supplementary Note 5). Figure 4a shows how we illuminate the dimer with a focused continuous-wave (CW) laser. To achieve the largest possible modulation, we again apply a voltage to allow the two particles to snap-to-contact and achieve a situation where the particles are separated by a mere 1.5-nm-thick EBID layer. We subsequently apply a time-modulated bias to dynamically compress the gap and modulate the scattered light from the particle pair. Figure 4b shows dark-field light scattering spectra taken from this dimer before (blue) and after (red) snap-to-contact. When the particle spacing is 30 nm, the overlapping BDP and ADP resonances produce a broad scattering peak around 1.75 eV, in agreement with the simulated and experimental EELS spectra (Figs. 1 and 2). In contrast, the spectrum taken after the snap-to-contact clearly shows how the BDP has nicely separated from the ADP mode. The spectral location of the BDP mode (1.23 eV) is consistent with an initial gap size around 1.5 nm before a bias is applied. In this situation, we can exploit the high sensitivity of the BDP mode to the particle spacing to achieve electrical modulation.

Upon application of a 5 V sinusoidal bias at 13 MHz, we observe a 5% scattering intensity modulation when we direct 1.27 eV photons to the dimer. This is quite substantial given that the illumination spot size (~10^6^ nm^2^) is significantly larger than the geometrical area of the dimers (~1.6 × 10^4^ nm^2^). We found that the introduction of the EBID layer facilitated robust operation for 7.8 × 10^9^ switching events. Other research where closely-spaced metallic nanostructures are placed on optical waveguides^38^ or where particles are placed above a reflective mirror^39,40^ show that stronger light-matter interaction can be achieved in such geometries. Whereas these configurations are less amenable to EELS studies, they open exciting opportunities to achieve signal modulation with an improved modulation depth.

The time evolution of the modulated signal is plotted as the magenta curve in Fig. 4c. To ensure, that the modulation is linked to the excitation of the BDP mode, we also measured the scattered light at a photon energy of 1.91 eV, away from the BDP resonance. At this frequency, no modulation was observed (gray curve in Fig. 4c). To probe the ultimate operation frequency limits of this modulator, we also simulated the properties of an Au nanoparticle pair without the nitride beams and found that resonance frequencies in the GHz range are achievable.

## Discussion

We have demonstrated dynamic electromechanical tuning of a strongly-coupled plasmonic dimer. EELS measurements with in situ biasing reveal the modal dispersion and spatial distribution of coupled plasmon modes at various gap sizes, enabling us to clearly differentiate BDP and ADP modes as a result of strong coupling. In the near-contact regime, the extreme tunability with gap size (250 meV/nm) of the BDP mode was then used to realize low-power, high-speed light intensity modulation. We anticipate that our results open up a promising route toward dynamic modulation of metasurfaces, chipscale optoelectronic switches, and quantum plasmonic systems. In such systems, the EBID materials can be replaced by designer dielectrics or molecules with unique optical responses that can e.g. be deposited using advanced chemical self-assembly approaches.

## Methods

### Electron energy loss spectroscopy simulations

The EELS simulations are performed using the Metallic NanoParticles Boundary Element Method (MNPBEM) toolbox for MATLAB^41,42^. The toolbox uses the boundary element approach to solve Maxwell’s equations in the presence of an inhomogeneous dielectric environment and electron beam excitation. In order to accurately model the optical response of our electromechanical dimer system, we import the experimental geometry of the dimer as obtained from a TEM image analysis. From the high-resolution TEM images, we trace out the top-view boundaries of our gold dimer and import them into the MNPBEM toolbox. This 2D geometry is extruded to a thickness of 50 nm (thickness of deposited gold layer) to construct a 3D object. The Si_3_N_4_ membrane is modeled using known literature values^43,44^.

### Sample fabrication

First, we deposit 60 nm of Si_3_N_4_ on the rear side of a standard 200 µm × 200 µm, 50-nm-thick Si_3_N_4_ TEM membrane (Protochips: E-AEL01-LN) using plasma-enhanced chemical vapor deposition (PECVD) to compensate the intrinsic internal mechanical stress in the membrane. Next, we fabricate two 50-nm-thick gold electrical actuator pads on the front side of the membrane using electron-beam lithography, metal deposition, and lift-off. Then, we use focused-ion beam (FIB) milling to pattern the gold-coated Si_3_N_4_ membrane into two free-standing cantilevers with connected gold nanodisks on the center of a connection bridge. In order to form separated cantilevers with a Au nanodisk, we flip over the sample and cut the center of the connection bridge from the rear side. By doing this, we get the sharpest gap features which would not be the case by milling from the front side.

### Electron energy loss spectroscopy (EELS)

#### Measurements

The EELS measurements and STEM imaging are performed with a FEI Titan TEM equipped with a monochromator and probe corrector. The microscope is operated in monochromated scanning TEM (STEM) mode at an acceleration voltage of 300 kV, providing a spot size of approximately 0.5 nm and an energy resolution of 0.1 eV (measured as the full-width at half-maximum of the zero-loss peak). The microscope is equipped with a GIF Tridiem electron energy-loss spectrometer and the Gatan DigiScan acquisition system. It can record an entire EELS intensity map in 10 to 20 min, depending on the number of pixels. We use a C3 aperture size of 30 μm, a camera length of 38 mm, an entrance aperture of 2.5 mm and a spectral dispersion of 0.01 eV per pixel in the EELS measurements. In addition, we utilize the automatic drift and dark current correction function included in the acquisition system. The individual EELS spectrum of the EELS intensity maps (with pixel sizes typically of 2–2.5 nm) are recorded with acquisition times of approximately 1 ms.

#### Post-processing of EELS data

To minimize the impact of monochromator drift during EELS acquisition, each EELS spectrum in the EELS data matrix is normalized to the zero-loss peak. Afterwards, the zero-loss peak of each EELS spectrum is removed using a Lucy-Richardson deconvolution algorithm, which sharpens the EELS data by increasing the energy resolution by a factor two approximately. We determine the number of deconvolution iterations following the procedure introduced in a previous work^45^. Briefly, a Voigt function is fitted to the zero-loss peak of the summed EELS data matrix (creating one spectrum for the entire matrix) in the energy range −1 to 1 eV. We implement a stopping criterion, which terminates the deconvolution algorithm when the change in the reconstructed spectrum is less than 2%. This criterion leads to between 15 to 20 deconvolution iterations. Equipped with a Voigt zero-loss profile and a fixed number of iterations, we perform the deconvolution algorithm on each spectrum in the EELS data matrix. The resonant EELS intensity maps shown in this paper depicts the summed EELS signal in a 0.10 eV spectral window centered at the resonance energies. The EELS signal from inside the nanoparticles are noisy and therefore removed in the map depictions. To detect the pixels inside the nanoparticles, we perform image analysis using the Image Processing Toolbox in MATLAB.

To show that our results are independent of the chosen data analysis method, we have also removed the zero-loss peak without using deconvolution techniques. We tested two additional methods to reconstruct the zero-loss peak: (*i*) the reflected-tail method, where the negative energy part of the zero-loss peak is mirrored around the zero-energy point to reconstruct the zero-loss peak, or (*ii*) fitting of a power-law function in the energy range 0.4 to 0.8 eV to reconstruct the background signal. Both of these approaches provide similar results to the deconvolution approach, but the latter is superior in terms of energy resolution and noise reduction.

### Energy-dispersive X-ray spectroscopy (EDS)

The energy-dispersive X-ray spectroscopy (EDS) measurements are performed with the same probe-corrected FEI Titan TEM as the EELS measurements. The microscope is operated in non-monochromated STEM mode at an acceleration voltage of 300 kV and is equipped with an Oxford INCAx silicon drift detector. The EDS acquisition is performed in the 0–20 keV spectral range with a pixel time of 0.5 ms, providing a frame time of 131.1 s (512 × 512 resolution). Typically, 20 frames are acquired for accurate elemental quantification. To enhance X-ray detection, the sample is titled 15 degrees. The data acquisition and analysis are performed using the INCA software platform.

### Optical characterization

#### Confocal dark-field spectroscopy

We use a confocal microscope (Nikon C2 Plus confocal microscope) with a dark-field module. The light from a tungsten-halogen lamp illuminates the sample through a dark-field condenser and a 100× objective lens (Nikon LU Plan Fluor 100× BD). The scattered light from the gold nanodisk dimer is collected by the same objective lens and the signal is transferred by an optical fiber connected to a spectrometer (SpectraPro, Acton 2300i). We use a Si array detector (Princeton Instruments, Pixis 1024) for the visible range measurements and an InGaAs array detector (Princeton Instruments, 7498-0001) for the near-infrared range measurements.

#### Time-resolved light modulation measurement

For the light intensity modulation experiments, we focus a 980 nm CW laser onto the Au dimer with a 100× objective lens (Nikon LU Plan Fluor 100× BD). The scattered light is collected by the same objective lens and fed into a confocal optical fiber which is connected to a Si avalanche photodiode (Micro Photon Devices). 13 MHz, 5 V, 50% duty cycle voltage square wave generated from a synthesized clock generator (SRS, CG635) actuate the dimer structure as well as trigger the time-correlated single photon counting module (PicoQuant, PH 300). The signal is sampled by a total of 7.8 × 10^9^ pulse trains. The data are averaged by binning every 4 ns.

## Supplementary information

Supplementary Information

## Data Availability

The data that support the plots within this paper and other findings of this study are available from the corresponding author upon reasonable request.

## References

[1] Baumberg JJ, Aizpurua J, Mikkelsen MH, Smith DR (2019). Extreme nanophotonics from ultrathin metallic gaps. Nat. Mater..

[2] Ciracì C (2012). Probing the ultimate limits of plasmonic enhancement. Science.

[3] Savage KJ (2012). Revealing the quantum regime in tunnelling plasmonics. Nature.

[4] Halas NJ, Lal S, Chang WS, Link S, Nordlander P (2011). Plasmons in strongly coupled metallic nanostructures. Chem. Rev..

[5] Prodan E, Radloff C, Halas NJ, Nordlander P (2003). A hybridization model for the plasmon response of complex nanostructures. Science.

[6] Nordlander P, Oubre C, Prodan E, Li K, Stockman MI (2004). Plasmon hybridization in nanoparticle dimers. Nano Lett..

[7] Su K (2003). Interparticle coupling effects on plasmon resonances of nanogold particles. Nano Lett..

[8] Romero I, Aizpurua J, Bryant GW, García De Abajo FJ (2006). Plasmons in nearly touching metallic nanoparticles: singular response in the limit of touching dimers. Opt. Express.

[9] Khurgin J, Tsai W-Y, Tsai DP, Sun G (2017). Landau damping and limit to field confinement and enhancement in plasmonic dimers. ACS Photonics.

[10] Mortensen NA, Raza S, Wubs M, Søndergaard T, Bozhevolnyi SI (2014). A generalized non-local optical response theory for plasmonic nanostructures. Nat. Commun..

[11] Raza S, Bozhevolnyi SI, Wubs M, Asger Mortensen N (2015). Nonlocal optical response in metallic nanostructures. J. Phys. Condens. Matter.

[12] Yan W, Wubs M, Asger Mortensen N (2015). Projected dipole model for quantum plasmonics. Phys. Rev. Lett..

[13] Kadkhodazadeh S, Wagner JB, Kneipp H, Kneipp K (2013). Coexistence of classical and quantum plasmonics in large plasmonic structures with subnanometer gaps. Appl. Phys. Lett..

[14] Schuck PJ, Fromm DP, Sundaramurthy A, Kino GS, Moerner WE (2005). Improving the mismatch between light and nanoscale objects with gold bowtie nanoantennas. Phys. Rev. Lett..

[15] Esteban R, Borisov AG, Nordlander P, Aizpurua J (2012). Bridging quantum and classical plasmonics with a quantum-corrected model. Nat. Commun..

[16] Scholl JA, García-Etxarri A, Koh AL, Dionne JA (2013). Observation of quantum tunneling between two plasmonic nanoparticles. Nano Lett..

[17] Miller DAB (2017). Attojoule optoelectronics for low-energy information processing and communications. J. Light. Technol..

[18] Duan H, Fernández-Domínguez AI, Bosman M, Maier SA, Yang JKW (2012). Nanoplasmonics: classical down to the nanometer scale. Nano Lett..

[19] Nelayah J (2007). Mapping surface plasmons on a single metallic nanoparticle. Nat. Phys..

[20] Polman A, Kociak M, García de Abajo FJ (2019). Electron-beam spectroscopy for nanophotonics. Nat. Mater..

[21] Raza S (2016). Electron energy-loss spectroscopy of branched gap plasmon resonators. Nat. Commun..

[22] Raza S (2015). Multipole plasmons and their disappearance in few-nanometre silver nanoparticles. Nat. Commun..

[23] Scholl JA, Koh AL, Dionne JA (2012). Quantum plasmon resonances of individual metallic nanoparticles. Nature.

[24] Di Martino G, Tappertzhofen S, Hofmann S, Baumberg J (2016). Nanoscale plasmon-enhanced spectroscopy in memristive switches. Small.

[25] Emboras A (2016). Atomic scale plasmonic switch. Nano Lett..

[26] Schoen DT, Holsteen AL, Brongersma ML (2016). Probing the electrical switching of a memristive optical antenna by STEM EELS. Nat. Commun..

[27] Holsteen AL, Raza S, Fan P, Kik PG, Brongersma ML (2017). Purcell effect for active tuning of light scattering from semiconductor optical antennas. Science.

[28] Haffner C (2019). Nano–opto-electro-mechanical switches operated at CMOS-level voltages. Science.

[29] Cencillo-Abad P, Ou JY, Plum E, Zheludev NI (2017). Electro-mechanical light modulator based on controlling the interaction of light with a metasurface. Sci. Rep..

[30] Shaltout AM, Shalaev VM, Brongersma ML (2019). Spatiotemporal light control with active metasurfaces. Science.

[31] Zheludev NI, Kivshar YS (2012). From metamaterials to metadevices. Nat. Mater..

[32] Lin X (2018). All-optical machine learning using diffractive deep neural networks. Science.

[33] De Leon NP, Lukin MD, Park H (2012). Quantum plasmonic circuits. IEEE J. Sel. Top. Quantum Electron..

[34] Yan W, Faggiani R, Lalanne P (2018). Rigorous modal analysis of plasmonic nanoresonators. Phys. Rev. B.

[35] Van Dorp WF, Hagen CW (2008). A critical literature review of focused electron beam induced deposition. J. Appl. Phys..

[36] Randolph SJ, Fowlkes JD, Rack PD (2006). Focused, nanoscale electron-beam-induced deposition and etching. Crit. Rev. Solid State Mater. Sci..

[37] Lee EK (2015). Resonant light scattering from a single dielectric nano-antenna formed by electron beam-induced deposition. Sci. Rep..

[38] Emboras A (2016). Atomic scale plasmonic switch. Nano Lett..

[39] Baumberg JJ, Aizpurua J, Mikkelsen MH, Smith DR (2019). Extreme nanophotonics from ultrathin metallic gaps. Nat. Mater..

[40] Tserkezis C (2015). Hybridization of plasmonic antenna and cavity modes: extreme optics of nanoparticle-on-mirror nanogaps. Phys. Rev. A.

[41] Hohenester U, Trügler A (2012). MNPBEM - A Matlab toolbox for the simulation of plasmonic nanoparticles. Comput. Phys. Commun..

[42] García de Abajo FJ, Howie A (2002). Retarded field calculation of electron energy loss in inhomogeneous dielectrics. Phys. Rev. B.

[43] Luke K, Okawachi Y, Lamont MRE, Gaeta AL, Lipson M (2015). Broadband mid-infrared frequency comb generation in a Si3N4 microresonator. Opt. Lett..

[44] Palik, E. D. *Handbook of Optical Constants of Solids II*. (Academic Press, 1998).

[45] Bellido EP, Rossouw D, Botton GA (2014). Toward 10 meV electron energy-loss spectroscopy resolution for plasmonics. Microsc. Microanal..

