# Supplementary material for: Nanoelectromechanical modulation of a strongly-coupled plasmonic dimer
Source: Nat Commun. 2021 Jan 4;12:48. doi: 10.1038/s41467-020-20273-2 (PMC7782521; doi:10.1038/s41467-020-20273-2)
Supplement: Supplementary file 1 — Supplementary Information [file 41467_2020_20273_MOESM1_ESM.pdf]

Supplementary Information

Nanoelectromechanical modulation of a strongly-coupled plasmonic dimer

Jung-Hwan Song[1†], Søren Raza[2*†], Jorik van de Groep[1], Ju-Hyung Kang[1], Qitong Li[1], Pieter G. Kik[3], and Mark L. Brongersma[1*]

[1]Geballe Laboratory for Advanced Materials, Stanford University, Stanford, CA 94305, USA.

[2]Department of Physics, Technical University of Denmark, DK-2800 Kongens Lyngby, Denmark.

[3]CREOL, The College of Optics and Photonics, University of Central Florida, Orlando, FL 32816, USA.

[†]These authors contributed equally to this work.

[*]brongersma@stanford.edu, sraz@dtu.dk

Supplementary Note 1: Eigenmode identification for the Au dimer

We perform three-dimensional eigenmode simulations in the frequency domain using a commercial software package (COMSOL). An add-in called "QNMEig" is used in order to properly take the dispersive properties of gold into account[1]. The permittivity of gold[2] is fitted by a Drude model, where we set the background relative permittivity, angular plasma frequency, and damping rate to be -10, 1.4×10^{16} Hz , and 1×10^{14} Hz, respectively. The simulation domain is set as an air (n = 1) sphere with a 400 nm radius surrounded by a 200-nm-thick perfect matching layer (PML). The Au nanoparticle dimer structure on the Si_3N_4 cantilever is modeled by two Au nanodisks on a free-standing, 110-nm-thick Si_3N_4 slab square (n = 2) with 300-nm-wide side length. We include the EBID material when the gap is smaller than 2 nm by superposing the Au nanodisk dimer at the center with a 20-nm-wide and 50-nm-thick dielectric cuboid (n = 1.42+0.11i). Supplementary Figure 1a shows the resonance frequencies of all the eigenmodes as a function of gap size, while Supplementary Fig. 1b-g and Supplementary Fig. 2a-f list the spatial distributions of the electric field and optically-induced charge density of each eigenmode. From 1 to 1.8 eV, we find coupled dipole resonances oscillating along either the x- or the y-direction. Due to the closer proximity of the dipole charges, the coupling between the x-oriented dipoles is stronger than for y-oriented dipoles, resulting in larger frequency splitting. The bonding vertical quadrupole mode (BVQP) and anti-bonding vertical quadrupole mode (AVQP) are found at 2.25 and 2.31 eV, respectively. These modes hardly shift with gap size changes from 30 to 0.5 nm. We identify the third lowest-order peak in Fig. 2a-c of the main manuscript to be the AVQP mode due

to the proximity of the AVQP eigenfrequency to the peak location and the visibility of the peak in the center EELS spectra for gap sizes smaller than 1.5 nm.

Supplementary Note 2: Electromechanical gap size control

To study the evolution of plasmon resonances as a function of gap size, we apply an external bias to the gold actuator pads on each of the cantilever arms to generate an electrostatic attraction force. Measuring the shortest distance between the two gold nanodisks in the STEM images, we plot the gap size as a function of bias voltage in Supplementary Fig. 3. From 0 to 30 V, the gap size shows a nearly monotonic decrease from 33 nm to 21 nm. From 30 V, the dimer hardly shows any motion. Reaching 60 V, the particles suddenly snap-to-contact and the gap size reduces to 1.5 nm. At this gap size, the dimer is already in mechanical contact through the EBID material. The compressibility of the EBID material affords fine control over the particle spacing.

Supplementary Note 3: Local electron excitation intensity of anti-bonding radial breathing mode

We plot the spatial EELS map of AVQP mode at 21 nm (Supplementary Fig. 4a) and 0.9 nm (Supplementary Fig. 4b). As discussed in Fig. 2 of the main manuscript, this mode barely shifts and a small 0.02 eV blue-shift is observed during the gap size change from 21 nm to 0.9 nm. The spatial profile also shows no perceptible changes during the gap size variation, consistent with the expectation for the AVQP mode.

Supplementary Note 4: Effect of fabrication imperfections on the gold dimer resonance properties

To clarify the reason for the deviation of the simulated resonance sensitivity to the gap size (70 meV/nm) from that observed in experiment (250 meV/nm), we investigate the dependence of the eigenfrequencies of the BDP and ADP modes on gap size for different fabrication imperfections. Fabrication imperfections can lead to final Au particle shapes that deviate from the intended circular nanodisks of a constant height that were simulated in the eigenmode calculations. For very small gap sizes and the BDP mode excitation, the fields redistribute to effectively concentrate light in the gap. As a result, a high sensitivity for the actual gap morphology can be expected. Supplementary Fig. 5a represents the top- and side-view of regular Au nanodisk dimer, while Supplementary Fig. 5b-c show those with laterally distorted particle shapes and those with non-uniform thickness, respectively, mimicking the fabricated structure (Fig. 1e). The eigenfrequency comparison shows the lateral shape distortion results in an enhanced interparticle coupling

(Supplementary Fig. 5d) between individual dipole resonance by almost a constant amount (50 meV) throughout the investigated gap sizes. We also study the effect of a non-uniform Au nanodisk thickness. The gold nanodisk thickness becomes slightly thinner near the gap interfaces, which is plausible for the FIB milling process for gap formation. The shift trend of the eigenfrequencies in the gold nanodisk dimer with non-uniform thickness matches well to the experimental results. For gap sizes larger than 1.5 nm, the eigenfrequencies of the gold dimer with non-uniform thickness agree well with those of the regular dimer. For gap sizes smaller than 1.5 nm, the eigenfrequency of the BDP mode in the gold nanodisk dimer with non-uniform thickness starts to deviate from that in the regular dimer, displaying an enhanced resonance sensitivity (200 meV/nm) similar to that observed in the experiments (250 meV/nm). The eigenfrequency of the ADP mode, on the other hand, does not show any noticeable deviation throughout all the examined gap sizes, which is also consistent to the experimental data (Fig. 2a-c).

Supplementary Note 5: Estimation of light intensity modulation energy

We estimate the energy required for modulation of our NEMS system using electromechanical simulations in COMSOL Multiphysics 5.5. Both silicon nitride beams are taken to be identical and all sizes are chosen to match the fabricated sample as best as possible. A 50-nm-thick gold layer is placed on top of the 110-nm-thick silicon nitride beams. The width of each beam is 500 nm, the length is 20 μm, and the gap between the beams is 600 nm. The beams are clamped at the ends. A region of 2 μm length in the center of each beam is taken to be gold-free as in the fabricated devices. The protruding silicon-nitride islands in the center of the beams are modelled as blocks with side lengths of 300 nm and the gold nanodisks have a diameter of 100 nm. The EBID bridge observed in the experiments is modelled as a 5×5 nm^2 wire with square cross section, connecting the nanodisks in the center of a 2-nm dimer gap. The material parameters for gold are taken as follows: density ρ_{Au} = 19.300 kg/m^3, Young's modulus E_{Au} = 78 GPa, and Poisson's ratio ν_{Au} = 0.44. For silicon nitride we use: ρ_{SiN} = 3.100 kg/m^3, E_{SiN} = 250 GPa, ν_{SiN} = 0.23, and relative DC permittivity ε_{SiN} = 7.5. For EBID we use: ρ_{EBID} = 2.000 kg/m^3, E_{EBID} = 30 GPa[3], ν_{SiN} = 0.3, and relative DC permittivity ε_{EBID} = 1.42.

By applying a DC voltage to the gold layers, an attractive static electric field is created between the beams, which induces a mechanical deflection towards each other. From the electric field distribution, we calculate the capacitance C of our device and subsequently the stored electric energy in charging the capacitor as $U_{el} = ½CV^2$. Supplementary Figure 6 shows the stored electric energy for different applied voltages. In addition, we numerically evaluate the stored strain energies in the beams and the EBID bridge due to their deformations. With our order-of-magnitude estimates for the EBID bridge size and Young's modulus, we find that the combined stored strain energy in both the beams and the EBID bridge does not exceed 0.1 aJ for an applied voltage of 5

V. Hence, the primary source of energy consumption in switching our device is electrical, with approximately 4 fJ at an applied voltage of 5 V. This suggests that shrinking and further optimization of the device geometry can lead to further reductions in the energy consumption.

Supplementary Figures

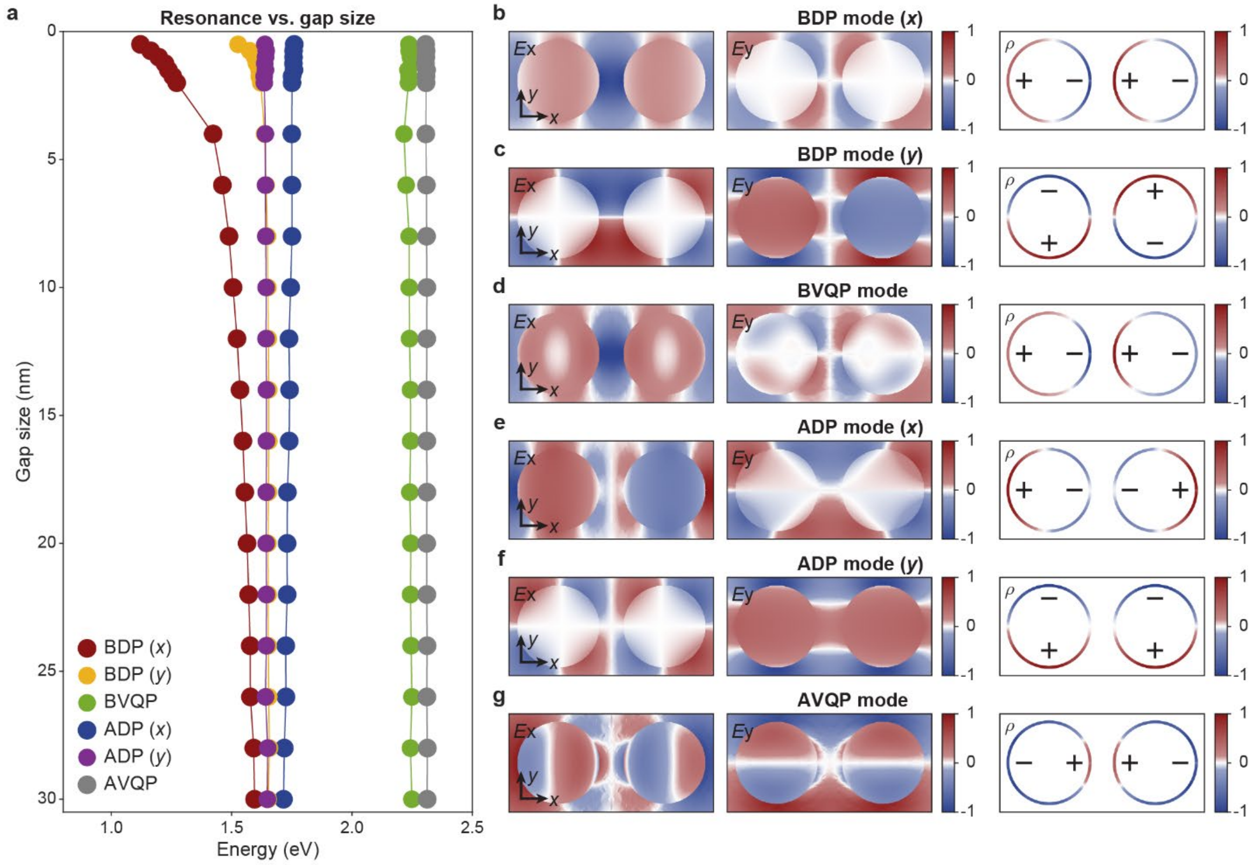

Supplementary Figure 1 | Eigenmodes for the Au nanoparticle dimer. a, Simulated eigenfrequencies of plasmonic resonances. **b-g,** Electric field components E_x, E_y, and optical charge density (ρ) profile of x-dipole BDP mode (**b**), y-dipole BDP mode (**c**), BVQP mode (**d**), x-dipole ADP mode (**e**), y-dipole ADP mode (**f**), and AVQP mode (**g**). We obtain the charge density by taking the divergence of the electric fields.

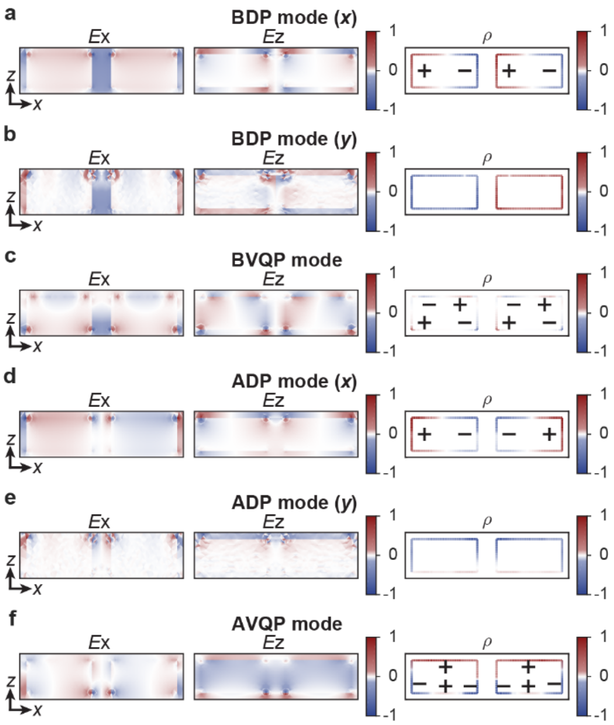

Supplementary Figure 2 | Eigenmodes for the Au nanoparticle dimer (cross section profile). a-f, Electric field components E_x, E_z, and optical charge density (ρ) profile of x-dipole BDP mode (**a**), y-dipole BDP mode (**b**), BVQP mode (**c**), x-dipole ADP mode (**d**), y-dipole ADP mode (**e**), and AVQP mode (**f**). We obtain charge density by taking the divergence of the electric field. For dipole modes oscillating along the y direction (**b**,**e**), the presented plane is the nodal plane and the field amplitude should all vanish in principle.

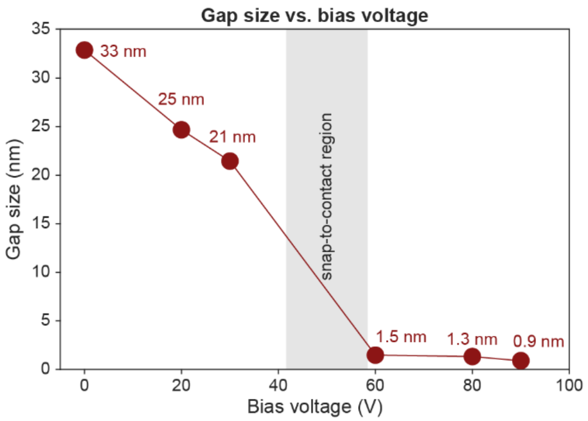

Supplementary Figure 3 | Gap size control by external bias. The measured gap size as a function of applied external voltage. The gap size is determined from the STEM images during the EELS characterization.

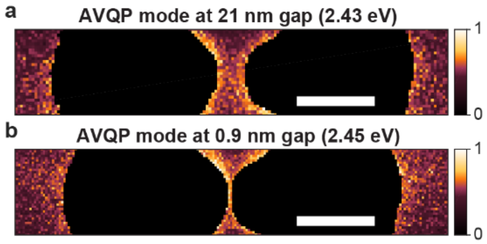

Supplementary Figure 4 | Local electron excitation intensity profile for the AVQP mode. a-b, The spatial EELS intensity profiles for the AVQP mode at gap sizes of 21 nm and 0.9 nm, respectively.

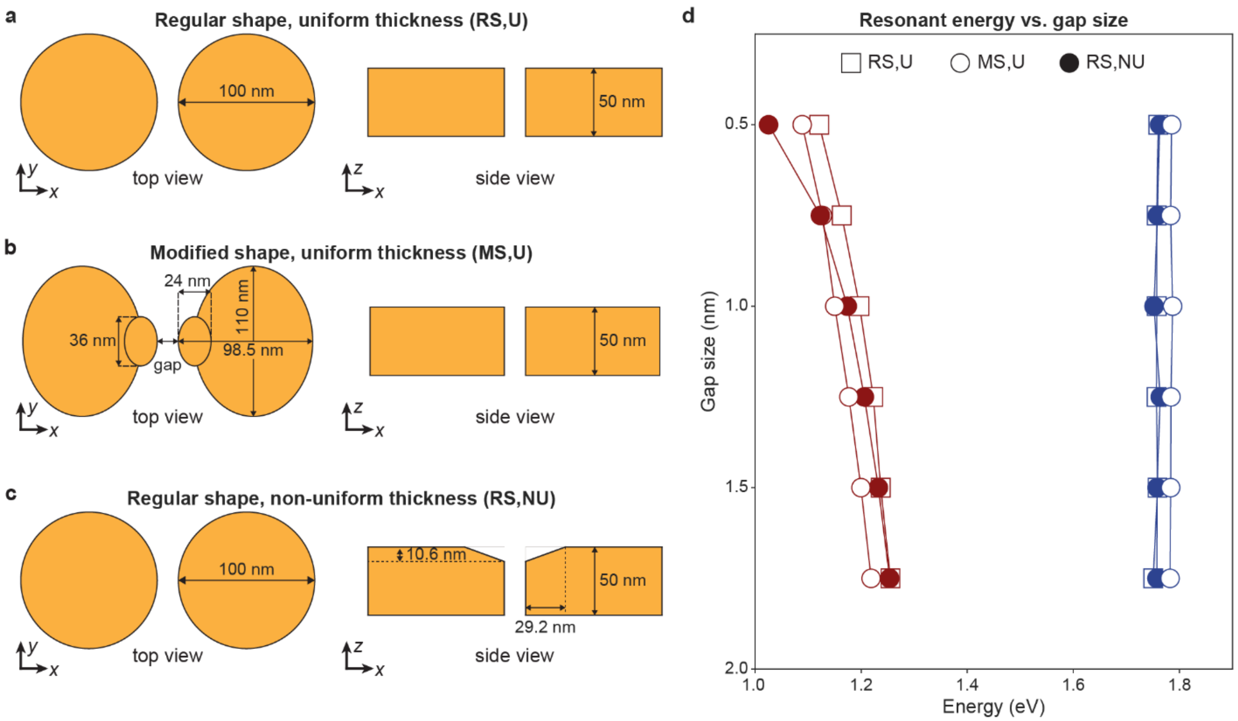

Supplementary Figure 5 | Effect of fabrication-induced imperfections on the optical resonance properties of the Au nanoparticle dimer. a, Top- and side-view of the intended Au nanoparticle dimer in the form of two closely-spaced nanodisks. **b,** Top- and side-view of a modified Au dimer, where the shape of each particle is approximated by two overlapping ellipsoidal particles **c,** Top- and side-view of a regular shaped Au dimer but non-uniform thickness. **d,** Simulated eigenfrequencies of the structures in **a**-**c** as a function of gap size.

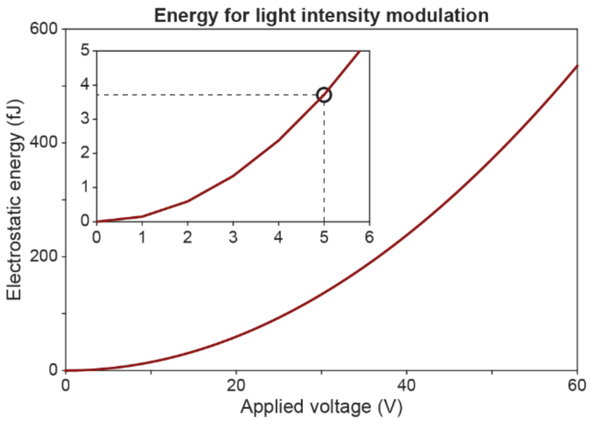

Supplementary Figure 6 | Electrostatic energy stored in NEMS platform. Calculated electrostatic energy based on simulated electric capacity of the NEMS platform. The inset highlights the voltage range near the amplitude of the input voltage (5 V).

Supplementary references

1. Yan, W., Faggiani, R. & Lalanne, P. Rigorous modal analysis of plasmonic nanoresonators. *Phys. Rev. B* **97**, 205422 (2018).
2. Johnson, P. B. & Christy, R. W. Optical Constants of the Noble Metals. *Phys. Rev. B* **6**, 4370–4379 (1972).
3. Marques, F. C. *et al.* Thermal expansion coefficient of hydrogenated amorphous carbon. *Appl. Phys. Lett.* **83**, 3099–3101 (2003).
